# Single-Cell Tracking on Polymer Microarrays Reveals the Impact of Surface Chemistry on *Pseudomonas aeruginosa* Twitching Speed and Biofilm Development

**DOI:** 10.1021/acsabm.0c00849

**Published:** 2020-11-06

**Authors:** Alessandro M. Carabelli, Marco Isgró, Olutoba Sanni, Grazziela P. Figueredo, David A. Winkler, Laurence Burroughs, Andrew J. Blok, Jean-Frédéric Dubern, Francesco Pappalardo, Andrew L. Hook, Paul Williams, Morgan R. Alexander

**Affiliations:** † Advanced Materials and Healthcare Technologies, School of Pharmacy, University of Nottingham, Nottingham NG7 2RD, U.K.; ‡ School of Computer Science, University of Nottingham, Nottingham NG8 1BB, U.K.; § Division of Molecular Therapeutics and Formulation, School of Pharmacy, University of Nottingham, Nottingham, NG7 2RD, U.K.; ∥ Biodiscovery Institute and School of Life Sciences, University of Nottingham, Nottingham NG7 2RD, U.K.; ⊥ Monash Institute of Pharmaceutical Sciences, Monash University, Parkville 3052, Australia; # La Trobe Institute for Molecular Science, la Trobe University, Bundoora 3083, Australia; ∇ CSIRO Data61, Pullenvale 4069, Australia

**Keywords:** polymers, high-throughput screening, Pseudomonas aeruginosa, twitching motility, biofilm

## Abstract

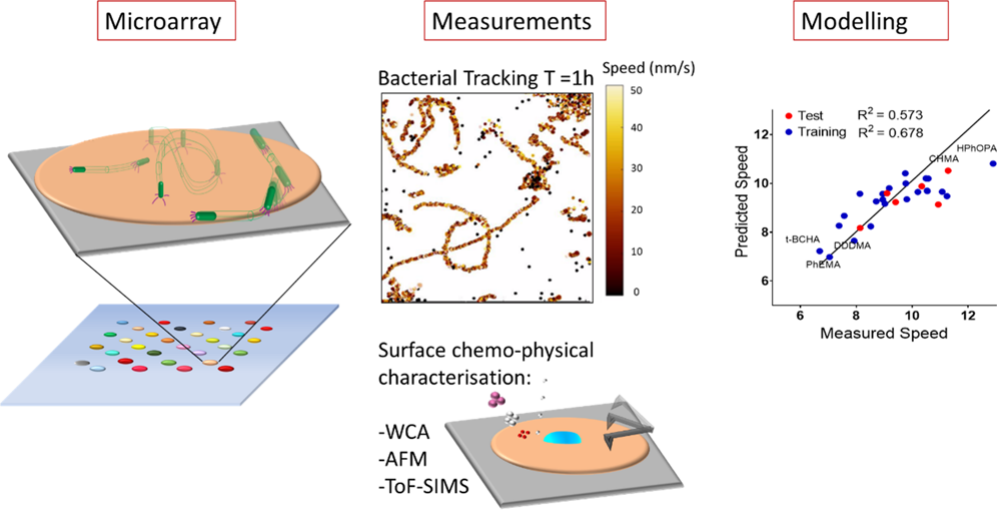

Bacterial biofilms exhibit up to 1000 times greater resistance to antibiotic or host immune clearance than planktonic cells. *Pseudomonas aeruginosa* produces retractable type IV pili (T4P) that facilitate twitching motility on surfaces. The deployment of pili is one of the first responses of bacteria to surface interactions and because of their ability to contribute to cell surface adhesion and biofilm formation, this has relevance to medical device-associated infections. While polymer chemistry is known to influence biofilm development, its impact on twitching motility is not understood. Here, we combine a polymer microarray format with time-lapse automated microscopy to simultaneously assess *P. aeruginosa* twitching motility on 30 different methacrylate/acrylate polymers over 60 min post inoculation using a high-throughput system. During this critical initial period where the decision to form a biofilm is thought to occur, similar numbers of bacterial cells accumulate on each polymer. Twitching motility is observed on all polymers irrespective of their chemistry and physical surface properties, in contrast to the differential biofilm formation noted after 24 h of incubation. However, on the microarray polymers, *P. aeruginosa* cells twitch at significantly different speeds, ranging from 5 to ∼13 nm/s, associated with crawling or walking and are distinguishable from the different cell surface tilt angles observed. Chemometric analysis using partial least-squares (PLS) regression identifies correlations between surface chemistry, as measured by time-of-flight secondary ion mass spectrometry (ToF-SIMS), and both biofilm formation and single-cell twitching speed. The relationships between surface chemistry and these two responses are different for each process. There is no correlation between polymer surface stiffness and roughness as determined by atomic force measurement (AFM), or water contact angle (WCA), and twitching speed or biofilm formation. This reinforces the dominant and distinct contributions of material surface chemistry to twitching speed and biofilm formation.

## Introduction

When bacterial cells colonize surfaces as biofilms, structured communities of sessile cells are enmeshed within a self-generated extracellular matrix that provides protection from antibiotics and host immune system clearance in humans and animals. Biofilms are responsible for chronic infections in implanted and indwelling medical devices such as catheters and for fouling of materials used in food processing, shipping, and oil recovery. Consequently, understanding how bacteria explore surfaces, interact with them, and form biofilms is important for the development of strategies for biofilm prevention and eradication.

Biofilm formation by bacterial pathogens such as *Pseudomonas aeruginosa* is thought to occur in four main stages: bacterial attachment to a surface, microcolony formation, biofilm maturation, and dispersal.^1^ The initial adhesion of bacteria to surfaces determines the final extent of biofilm formation.^2,3^ The flagella enable single bacterial cells to reach a surface by swimming through liquid environments, whereas type IV pili (T4P) are required for twitching motility employed by single cells to crawl or walk on surfaces.^4^ T4P pull the bacterial cell body forward by pilus extension and retraction through cycles of polymerization and depolymerization of the major pilin protein subunit PilA. Pili and flagella have also been shown to be involved in surface detachment and possibly in biofilm dispersal.^5^ They also mediate a form of social migration called swarming that enables *P. aeruginosa* cells to collectively colonize surfaces.^6,7^ In addition, bacterial populations mount cooperative responses to environmental signals through quorum sensing (bacterial cell-to-cell communication) mechanisms that orchestrate complex circuits to regulate virulence and biofilm formation.^8^

Early-stage surface interrogation involving flagella and T4P not only supports bacterial cell migration but is also involved in surface sensing and the decision to switch from the motile to the sessile state. *P. aeruginosa* cells display distinctive biological features as early as 20 min after inoculation onto a surface.^9−11^ Furthermore, an incubation time of ∼30 min without shear stress has been shown to be necessary before significant bacterial adherence to surfaces is observed. Prior to this time point, cells adhere to different surfaces with the same low adhesive force and do not proceed to later-stage biofilm formation. This initial 30 min period was interpreted as the cells sensing the surface and making the decision whether to adhere and become irreversibly attached or to leave the surface.^12^

Biofilm formation is also influenced by the T4P functioning as surface adhesins under flow conditions. Twitching motility contributes to cell aggregation and microcolony formation during early-stage biofilm development and to the structuring of biofilm architecture during later developmental stages.^7^*P. aeruginosa* secrete exopolysaccharide trails (Psl) when twitching on glass, guiding other cells along the trails and allowing bacteria to self-organize into microcolonies.^13^ Mutant *P. aeruginosa* strains unable to produce or retract their T4P form aberrant biofilms.^7,14^ Under certain growth conditions, T4P are essential, as *P. aeruginosapilA* mutants fail to form mature biofilms.^15^ These findings have highlighted the important roles of T4P and the twitching motility during the different stages of bacterial biofilm development.

Certain surface microtopographies have been reported to hinder T4P-driven *P. aeruginosa* surface migration, potentially impacting on microcolony formation and subsequent biofilm development.^16,17^ However, little attention has been paid to the contribution of the surface chemistry of materials. This is an important knowledge gap in our understanding of T4P-mediated surface sensing, twitching motility, and the role of material surface chemistry in controlling bacterial cell movement and biofilm formation.

Twitching motility can be evaluated by macroscopic and microscopic assays.^18^ By stabbing a bacterial inoculum through semisolid agar in a Petri dish, twitching can be observed as interstitial colony expansion occurring at the interface between the agar and polystyrene. Tracking single cells during twitching requires high-resolution microscopy to allow discrimination between the cell orientations characteristic of T4P-mediated walking and crawling.^5,18,19^

Polymers with intrinsic resistance to bacterial biofilm formation are an expanding area of biomaterial research as they offer an effective alternative to leachable antimicrobial coatings that have limited life and can induce bacterial resistance.^20−22^ High-throughput polymer microarray screening has been employed to simultaneously assess hundreds of materials for their resistance to biofilms of pathogenic bacteria^21,22^ and most recently fungi.^23^ The broad chemical diversity of readily available commercial acrylate monomers, including linear-, cyclic-, aromatic-, and heteroatom-substituted moieties, enabled the discovery of a class of weak amphiphilic polymers incorporating ester/amide and cyclic hydrocarbon groups. These inhibit bacterial biofilm formation and subsequently have been successfully employed as a coating on silicone urinary tract catheters.^24^ The precise mechanism by which these polymers prevent biofilm formation is not fully understood but involves the interplay between bacterial sensing and surface chemistry.^25−27^ It is likely to be different from the well-known effects of hydration on and in hydrophilic surfaces and its effect on bacterial surface attachment.^28,29^

Here, we describe the effect of surface chemistry on the twitching motility and subsequent biofilm formation by *P. aeruginosa*. We employed a polymer microarray and high-resolution time-lapse microscopy to simultaneously measure twitching motility on 30 chemically diverse methacrylate/acrylate polymers. Atomic force measurement (AFM) and water contact angle (WCA) measurements were made to better understand the contribution of material roughness, stiffness, and wettability. Partial least-squares (PLS) regression models describing the relationship between polymer surface chemistry and microbial responses allowed the identification of chemical moieties associated separately with twitching and later-stage biofilm development.

## Experimental Section

### Polymer Array Synthesis

Polymer microarrays were synthesized using methods previously described.^22^ To achieve improved optical clarity compared with previous polymer microarrays,^20,22^ monomer solutions were printed as 800 μm diameter spots directly onto a methacrylate-silanized coverslip and UV-cured (Figure S1). A methacrylate-terminated silane was employed to covalently link the polymer spots to the glass support. Coverslips were used as a support to enable the use of a high numerical microscope aperture (N.A. = 1.4) and low working distance (WD = 0.13 mm) objectives. Monomers were purchased from Sigma-Aldrich, Scientific Polymers, and Polysciences and printed onto methacrylate-silanized glass borosilicate coverslips (Gerhard Menzel, Braunschweig, Germany) (22 × 22 mm^2^, 0.16–0.19 mm thickness). To avoid delamination, glass was treated with oxygen plasma at a 1000 W incident LF (40 kHz) power for 5 min at a 0.09 mbar working pressure by using a Diener nano Plasma machine.^30^ Oxygen plasma treatment was used to activate the glass surface by exposing hydroxyl groups. Activated coverslips were methacrylate-silanized by immersion in 3-trimethoxysilylpropyl methacrylate (Sigma-Aldrich) 2% v/v at 50 °C under 1 atmosphere of argon for 16 h. Coverslips were then washed with acetone to remove agglomerated silanes and then placed under vacuum for 24 h before use. Monomer solutions were prepared using 5% v/v monomer and 95% v/v dimethyl sulfoxide (DMSO) with 1% w/v of initiator 2,2-dimethoxy-2-phenylacetophenone (DMPA) (Sigma-Aldrich). Sixty microliters of each solution was transferred into a 384-well microplate (Polypropylene) with a 120 μL volume capacity. Printing was performed by an XYZ3200 dispensing work station (Biodot) with steel pins (Array-It, 946-6B) (capacity of 2.4 nL of H_2_O). Contact printing of the monomers was performed in an environment of 45–50% humidity, 25 °C, and <2000 ppm of O_2_. Polymerization was achieved *in situ* by exposure of the printed slides to shortwave 365 nm UV light (density, 30 mW/cm^2^).

### High-Throughput Surface Characterization

Polymer arrays were characterized by AFM,^31^ WCA,^32^ and time-of-flight secondary ion mass spectrometry (ToF-SIMS)^33^ as previously described. ToF-SIMS measurements were conducted on an ION-ToF IV instrument operated using a monoisotopic Bi_3_^+^ primary ion source operated at 25 kV and in “bunched mode”. A 1 pA primary ion beam was rastered, and both positive and negative secondary ions were collected from an 8 × 8 mm^2^ area. Ion masses were determined using a high-resolution time-of-flight analyzer. The typical mass resolution (at *m*/*z* 41) was just over 6000. For data analysis using the SurfaceLab 7 software (IONTOF GmBH, Germany), specific spots associated with individual polymers on the microarray were extracted using the region of interest (ROI) ellipse selection tool, and the resulting mass spectrum was calibrated prior to peak assignment. [CH_3_]^+^, [C_2_H_5_]^+^, [C_3_H_7_]^+^, and [C_4_H_9_]^+^ were chosen as positive calibration ions, while [OH]^−^, [C_2_H]^−^, [C_3_]^−^, and [C_4_H]^−^ were chosen as negative.

WCA was measured using the sessile drop method on an automated Krüss DSA 100 instrument. A water drop with a volume of ∼400 pL was used. Force measurements were made on an Asylum MFP-3D AFM (Oxford Instruments, Asylum Research Inc., CA) in contact mode. The probe used was an RTESPA-300 probe (Bruker Nano Inc., CA) exhibiting a 30.6 N/m spring constant and a 314.47 kHz resonant frequency. One hundred and sixty-nine force curves were taken over three separate 60 μm^2^ areas per replicate. The standard used for AFM measurement reproducibility was a polystyrene substrate with a nominal Young’s modulus of 2.7 GPa (PSFILM-12M, Veeco, CA). The Derjaguin–Muller–Toporov (DMT) model was used to calculate Young’s modulus *via* the slope from the upper portion of the retraction curves to remove contributions by plasticity. Sample indentation was carried out under dry and liquid conditions after immersing the sample in 18.2 Ω·cm milliQ water for at least 5 min. AFM topographical analyses were taken using a Bruker Dimension Icon AFM (Bruker Nano Inc., CA) and using a PeakForce QNM mode for imaging. Bruker MSNL-F tips were used (silicon tip/nitride lever) with a 0.97 N/m spring constant (calculated *via* the Sader method) and a resonant frequency of 116.29 kHz. Areas of 1 × 1 μm^2^ were recorded. Samples were assessed under liquid conditions after being immersed in 18.2 Ω·cm milliQ water for at least 5 min. All data of the topographic images were corrected using Gwyddion, SPM Data Analysis software, *via* mean plane subtraction, row realignment, and removal of horizontal scars. Root-mean-square deviation (RMS) values for roughness were calculated using an average of >3 measurements.

### Bacterial Strains and Growth Conditions

*P. aeruginosa* PAO1 (Washington subline, Nottingham collection) and its isogenic *P. aeruginosa* Δ*pilA* in frame deletion mutant (this laboratory) were each grown at 37 °C in lysogeny broth (LB) with shaking at 200 rpm or on semisolid LB agar (2% w/v). To visualize bacterial cells, cells were transformed with a plasmid containing the red fluorescent protein mCherry by electroporation with the plasmid, pMMR.^34^ Cells that are no longer viable express this fluorescence since they require protein turnover to be maintained. Bacterial biofilm formation assays were conducted as previously described.^22^ Microarray coverslips were UV-sterilized and placed in a 4-well Petri dish containing an RPMI-1640 medium. After inoculation with mCherry-tagged *P. aeruginosa*, the microarrays were incubated for 24 h at 37 °C and shaken at 60 rpm. *P. aeruginosa* biofilm grown under these conditions displays some areas where single cells can be observed at the surface but also large areas encased in extracellular matrix, as shown in Figure S1c. Control microarray coverslips were also incubated in the same conditions without bacteria. After incubation, slides were rinsed with phosphate-buffered saline and then with distilled water for 5 min.

### Polymer Microarray Fluorescence Imaging

Images of control and bacteria-exposed coverslips were acquired using a GenePix Autoloader 4200AL (Molecular Devices) scanner with a 655–695 nm filter. Bacterial biofilm formation on each polymer spot was quantified by subtracting the background fluorescence of the control microarray coverslips from that exposed to bacteria. We measured the fluorescence signal (*F*) that indicated the total fluorescence signal per unit area on any spot on the array.

### Individual Cell Tracking and Quantification of Twitching Motility

Time-lapse imaging was achieved using a bespoke multimode microscope (Cairn Ltd.).^35^ Samples were examined at 37 °C using a Nikon Eclipse Ti inverted microscope using a 40×, NA = 1.4, WD = 0.13 mm oil objective. The microscope was fitted with an environmental chamber (Okolab) to regulate temperature and relative humidity. Images were acquired using an Orca-Flash 4.0 digital CMOS camera (Hamamatsu) every 2 min. Differential interference contrast (DIC) brightfield and widefield epifluorescence imaging was achieved using a single-channel white MonoLED (Cairns) light source. Experiments were conducted using a custom-designed single-well holder under the microscope in static conditions (Figure S2). The holder allows a microarray coverslip to be inserted and filled with 4 mL of culture medium. To avoid leaks, coverslips were sealed using a silicone- and halogen-free high-vacuum grease (Apiezon M).

To obtain bacterial tracking data, previously developed bespoke MATLAB scripts were employed.^35^ Instantaneous speed was calculated as a function of the displacement vector as shown in eq 1 where Δ*t* = *t*_*i*+1_ – *t*_*i*_, while the average speed was calculated according to eq 2 where *n* is the number of points in the track
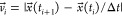
1
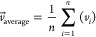
2*P. aeruginosa* T4P-driven twitching motility can adopt one of the two distinct modes: flat “crawling”, where the cell is lying flat on the surface, or upright “walking”, where the cell is in contact with the surface at only one pole. Walking cells have been described as moving faster than crawling cells.^5^ We adapted the method developed by Rodesney et al. to measure walking and crawling distributions.^36^ As a proxy readout for tilting, the projected aspect ratio in each frame was considered. A newly divided cell lying flat on the surface has an aspect ratio of about 2. Therefore, a projected aspect ratio less than 2 indicates unambiguously that a cell is tilting. Aspect ratios greater than 2 are also classified as cells lying flat. This may also include cells undergoing division that are in contact with the surface at only one pole in a tilting position. Visual inspection of frames showed that the fraction of cells undergoing division was <2% and the contribution of this error was evaluated as low. For each frame, cells were unambiguously computed as either tilted up or lying flat. For each polymer, we considered the average tilting fraction as the average of the tilting fractions of cells over the entire duration of the experiment.

### Partial Least-Squares Regression (PLS) Analysis

Correlations between ToF-SIMS spectra and bacterial twitching motility speed and biofilm formation were analyzed using PLS regression. In total, 640 positive and 892 negative ions were selected to form the peak list. Both positive and negative ion peak intensities were then normalized to the respective total secondary ion counts to remove the influence of primary ion beam fluctuation. The positive and negative ion intensity data were merged into one data matrix that was mean-centered and square-root-mean-scaled prior to analysis. PLS analysis was carried out using PLS Toolbox 5.2 software (Eigenvector). The data set was randomly split into a training group, containing 75% of the samples, and a test set, containing the remaining 25% of the samples. The test set was selected by ranking the samples by cell number and randomly selecting 25% of samples from the lowest 25%, middle 50%, and highest 25% of samples. The training set was formed from the remaining samples. PLS models were constructed using latent variables corresponding to local minimum or inflection points in the root-mean-square error of cross-validation (RMSECV) curve. The accuracy of the PLS models was evaluated by predicting the properties of the test set not used to generate the models. The final PLS model was constructed using the latent variable, whereby the *R*^2^ and root-mean-square error (RMSE) values for the test were a maximum and close to the values of the training set.

### Statistics

All experiments were conducted as three independent replicates (*N* = 3). Bacterial motility studies were conducted considering more *n* > 100 tracks for each polymer. Analysis of variance (ANOVA) test and Tukey’s multiple comparison were used to compute statistical analysis of experiments with multiple groups. piBUMA was chosen as control for statistical analysis of twitching speed because it was found to be in the middle of the experimental twitching speed range and because it had been extensively studied previously.^21,22^

## Results and Discussion

### Sample Preparation and Characterization

**Figure 1:**
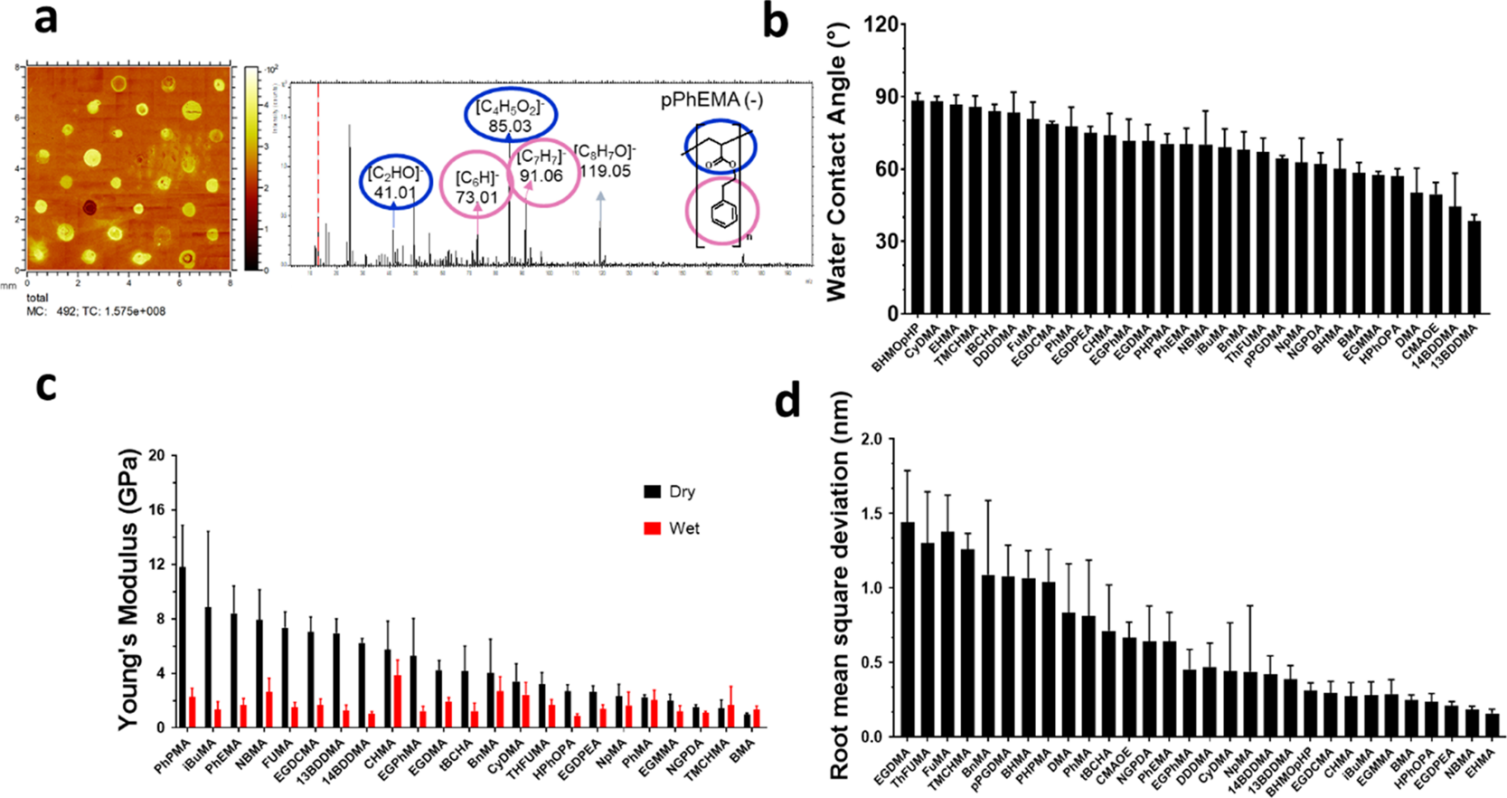
Polymer microarray characterization: (a) ToF-SIMS image of the total secondary ion intensity from the polymer microarray and a representative negative polarity spectrum from poly(2-phenylethyl methacrylate) pPhEMA with assigned peaks and structural fragments denoted with pink and blue circles; (b) WCA measurements for each microarray polymer spot; (c) Young’s modulus under dry and wet conditions for each polymer ranked from the highest to lowest Young’s modulus under dry conditions. Some polymers such as poly(decyl methacrylate) (pDMA), poly(benzhydryl methacrylate) (pBHMA), poly(propylene glycol) dimethacrylate pPGDMA, poly(1,10-decanediol dimethacrylate) (pDDDMA), poly(ethylhexyl methacrylate) (pEHMA), poly(caprolactone 2-(methacryloyloxy)ethyl ester) (pCMAOE), and poly(2,2-bis[4-(2-hydroxy-3-methacryloxypropoxy)) phenyl]propane (pBHMOPhP) have been excluded because they adhered to the AFM probe tip in dry and wet conditions preventing accurate measurements; (d) determination of the root-mean-square roughness (nm) at 1 μm^2^ from the surface under liquid conditions. Values are the mean of three images taken over three different samples. The error bars equal ± 1 SD (*N* = 3). Plots are ranked in separate orders.

The library of 30 methacrylate/acrylate monomers contained functional groups such as linear and cyclic hydrocarbons and hydroxyl groups previously shown to strongly influence *P. aeruginosa* biofilm formation.^21,25,26^ Their nomenclature, chemical structures, and sources are presented in Table S1. The polymer microarrays were characterized *via* ToF-SIMS, WCA for wettability measurements, and AFM for topographical characterization and Young’s modulus determination (Figure 1). ToF-SIMS imaging of the polymer microarray (Figure 1a) demonstrated that all 30 spots had been successfully printed without spreading or unintended monomer transfer between spots. A region of interest was assigned for each polymer spot for the acquisition of individual SIMS spectra (Figure 1a). Picoliter sessile drop WCA measurements were performed to probe surface wettability. The WCAs varied from 36 to 88° reflecting the chemical diversity of the microarray (Figure 1b). To obtain the elastic modulus, we used AFM in indentation mode (under both dry and wet conditions) on each polymer; dry measurements were consistent with previous observations on polymers.^37^Figure 1c shows that material compliance increases when hydrated (to various degrees), which we assume causes the drop in stiffness associated with water uptake (Figure S3). All materials exhibited smooth surfaces in wet conditions, with RMS roughness acquired from the AFM images of <2 nm in agreement with values previously reported by Hook et al.^22^

### Relationship between Surface Chemistry and Twitching Motility

**Figure 2:**
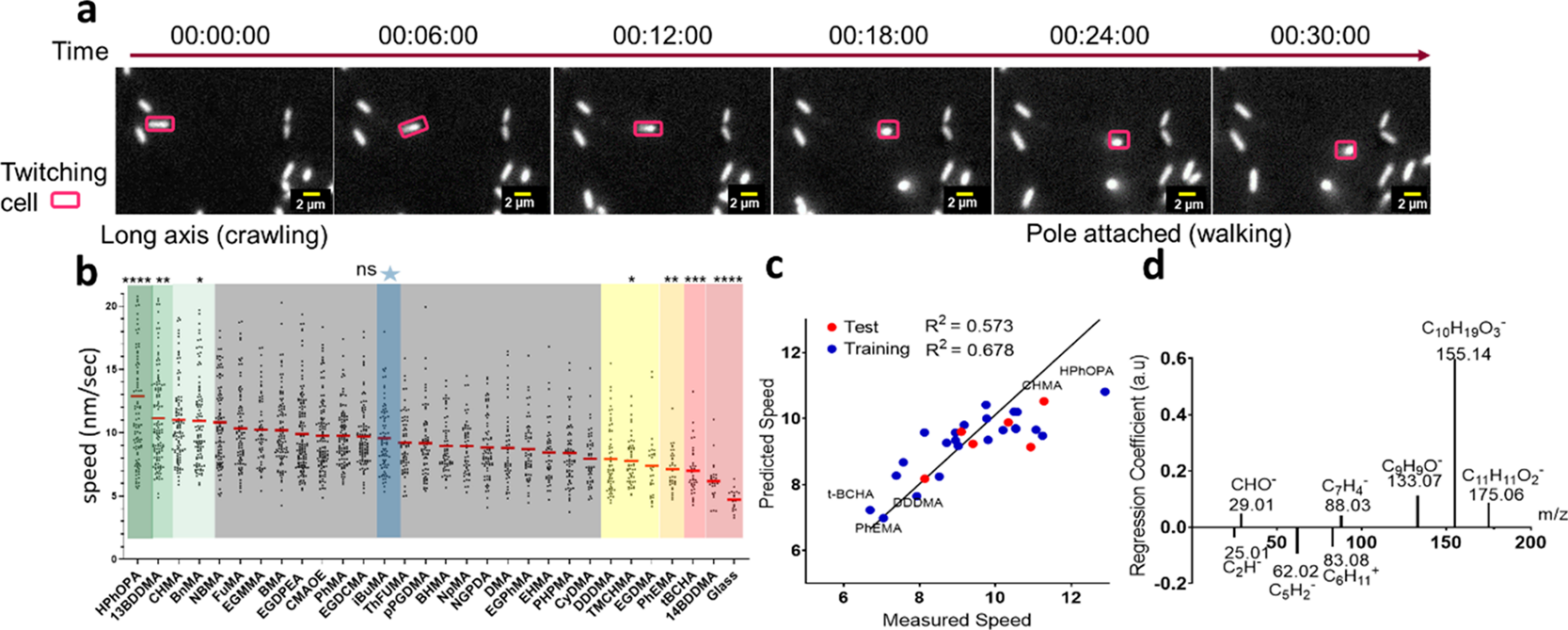
Relationship between polymer chemistry and *P. aeruginosa* twitching motility. (a) Time-lapse frames showing examples of *P. aeruginosa* twitching cells (red circles) on pHPhOPA obtained using epifluorescence microscopy. Scale bar, 2 μm; (b) scatter dot plot showing average twitching speeds of tracks of single *P. aeruginosa* cells on each polymer (*N* = 3). The mean is presented as a red line. Statistical differences are shown with different colors (gray, no-statistical-difference group). The blue star indicates the control used for multiple comparisons. Significance was determined by analysis of variance one-way ANOVA and Tukey’s post-test comparison for differences between the indicated samples. ****p < 0.001, ***p < 0.001, **p < 0.01, and *p < 0.05 are highlighted by different colors. (c) Predicted bacterial twitching average speed determined from the PLS regression model used to predict the biological performance of materials by correlating speed with the ToF-SIMS ions selected from the least absolute shrinkage and selection operator (LASSO) analysis (*R*^2^ = 0.686 and 0.573 and RMSE = 0.57 and 0.51 for the training and test data sets, respectively). This did not include glass; (d) regression coefficients (RCs) obtained from PLS regression analysis from latent variable 2. Ions with high and low RCs are shown.

To investigate the relationship between twitching motility and surface chemistry, *P. aeruginosa* was cultured in a custom-designed holder for the polymer microarray that allows simultaneous high-resolution imaging. Time-lapse imaging in conjunction with the automated stage movement allowed the collection of consecutive images of 100 × 100 μm^2^ area from each of the 30 polymers. Images were acquired from the focal plane near the surface every 2 min. Software was used to reconstruct tracks from the consecutive images providing sufficiently high temporal resolution to confidently measure the speed of twitching cells in the range of 3 and 21 nm/s. These limits corresponded to a minimum and maximum cell center of mass displacement between frames of 0.3 μm (2 pixels) and 2.5 μm (25% longer than the cell body length), respectively. This maximum displacement was ∼3 times greater than the instantaneous twitching speeds previously reported.^36^ The sampling frequency was too low to reconstruct tracks of individual swimming cells, which have speeds of ≥50 μm/s, much higher than our experimental maximum speed, leading them to be discarded in the track reconstruction analysis.^35^ The movement of *P. aeruginosa* cells on the surface was confirmed as twitching motility, and hence T4P-dependent, by comparing the wild-type *P. aeruginosa* strain with an isogenic Δ*pilA* mutant (Figure S4). Using data from this experiment, the speed, direction, and tilt of twitching wild-type *P. aeruginosa* cells can be compared with sessile bacterial cells for each polymer during the first hour post inoculation (Figure 2).

The speed of *P. aeruginosa* twitching on all microarray polymer surfaces showed a significant range for each polymer, likely arising from the various orientations that different motile cells adopted relative to the surface that we consider in detail later. Since the instantaneous speed values estimated during each track measured for cells on all polymers (Figure S5) show normal distributions, we used the mean speed for each track to represent the cell twitching speed, which is presented as a single data point in Figure 2b. This provides a large range of speeds for the many tracks on each polymer, although the highest and lowest mean track speeds for certain polymers across the library were statistically significantly different from an intermediate group of materials (Figure 2b). The lowest average twitching speed was observed on glass (4.7 ± 0.8 nm/s). Bacterial cells moved faster on average on poly(hydroxy-3-phenoxypropyl acrylate) pHPhOPA exhibiting an average speed of 12.9 ± 0.5 nm/s, whereas, on others, the cells migrated significantly slower with the lowest mean on a polymer observed on poly(1,4-butanediol dimethacrylate) p14BDDMA at an average speed of 6.1 ± 1.5 nm/s. One-way ANOVA and Tukey’s post-test comparison, as shown by different colors representing groups of polymers, revealed differences between the indicated samples (****p < 0.0001, ***p < 0.001, **p < 0.01, and *p < 0.05). Polymers within the same colored group in Figure 2b showed no statistical differences between them. We did not observe a change in twitching speed in response to cell density or exposure time (Figure S6).

To further investigate the structure–function relationship governing different bacterial twitching speeds on the polymers, we explored correlations between the surface chemistry and bacterial speed. Bacterial twitching responses to the polymeric library could not be correlated with any single SIMS ion, likely due to the number and diverse nature of the polymeric chemistries used in the study. Thus, PLS regression was used to investigate whether correlations exist between the surface chemistry of the polymers represented by all of the secondary ions detected by ToF-SIMS in the spectra of the polymers with twitching speed. A least absolute shrinkage and selection operator (LASSO) test was performed for feature selection to generate a sparse ToF-SIMS data set (Table S2).^38^ PLS models were then constructed using two latent variables corresponding to local minimum or inflection points in the root-mean-square error of cross-validation (RMSECV) curve. The data set was randomly split into training and test sets (75:25). The final PLS model was constructed using the latent variable, whereby the *R*^2^ value for the test was a maximum and close to the *R*^2^ value of the training set. The PLS regression (Figure 2c) successfully predicted twitching speeds, with a linear correlation (training *R*^2^ = 0.69, RMSE = 0.57; test *R*^2^ = 0.57, RMSE = 0.51) observed between predicted and experimental values. This relationship describes how T4P-driven twitching motility depends on polymer surface chemistry. Each ion was assigned a regression coefficient for the PLS model, and its size and sign provided insight into which molecular properties influenced the twitching speed (Figure 2d). Ions with higher coefficients included [CHO]^−^, [C_10_H_19_O]^−^, [C_9_H_9_O]^−^, and [C_11_H_11_O_2_]^−^. The [CHO]^−^ ion was produced by all polymer samples but with varying intensities. Other ions contributing strongly and positively to the twitching speed model were observed in the spectra of pHPhOPA, poly(cyclohexyl methacrylate) pCHMA, and poly(benzyl methacrylate) pBnMA, consistent with the high twitching speeds observed on these polymers. The molecular ion with the largest negative regression coefficient was [C_5_H_2_]^−^. It occurred with highest intensity in poly(1,10-decanediol dimethacrylate) pDDDMA, poly(trimethylcyclohexyl methacrylate) pTMCHMA, and poly(2-phenylethyl methacrylate) pPhEMA. The ion C_6_H_11_^+^ had the second largest negative regression coefficient. It occurred with relatively high intensity in the spectrum of poly(*tert*-butylcyclohexyl acrylate) p*t*BCHA, reflecting the low twitching speed observed on this polymer.

These results suggest a key influence of cyclic hydrocarbon pendant groups on twitching speeds. However, as disparate biological responses were observed on p*t*BCHA and pHPhOPA, which both contain cyclic hydrocarbons, it is likely that the controlling polymer characteristics of the biological material relationship are more complex, and elucidation would require further structure–performance studies involving synthesis of analogues including systematic variation of the polymer backbone.^26^

### Bacterial Crawling and Walking

**Figure 3:**
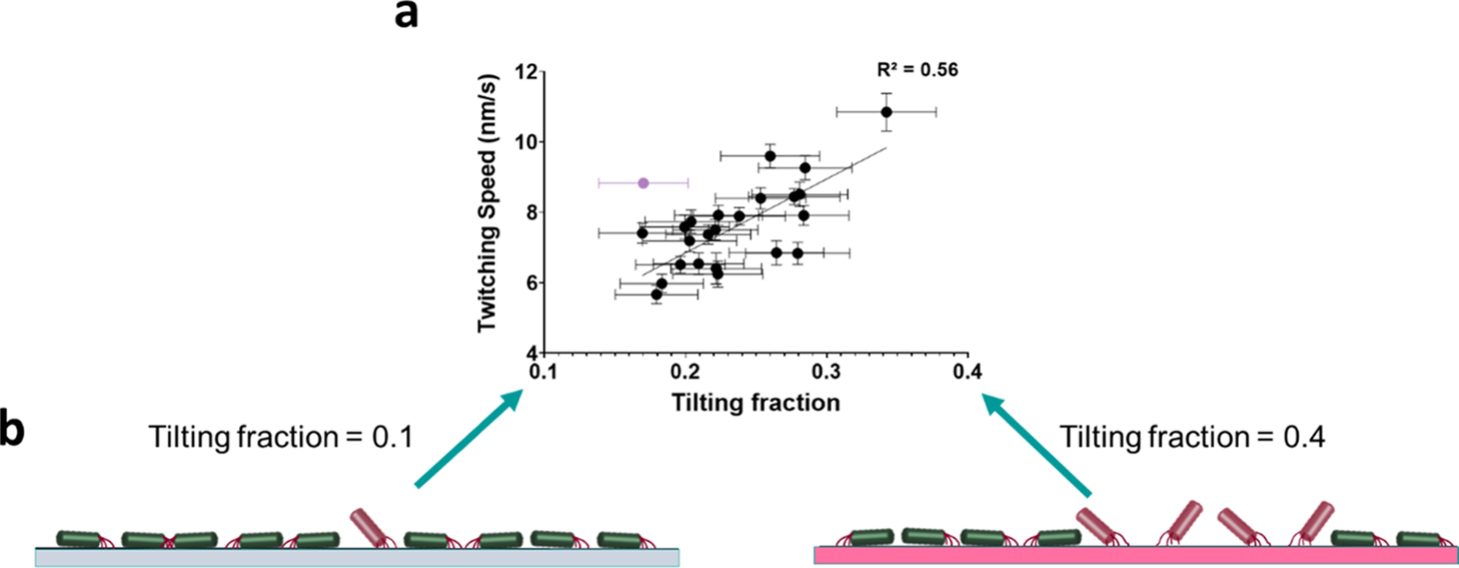
(a) Scatter plot showing the average twitching speed of cells as a function of the tilting fraction for the entire population of cells over 1 h of exposure to the different polymer surfaces. The violet data point corresponding to pFuMA had an absolute residual greater than 2 × SD of speed and so was omitted from the fit; (b) diagram depicting the tilting fractions on two different materials mediated by T4P localized at the cell pole.

The T4P-driven twitching motility in *P. aeruginosa* cells comprises two distinct modes: “crawling”, where the cell is oriented with the long axis of the rod-shaped body parallel to the surface; and more upright “walking”, where it is in contact with the surface at only one pole.^5^ In Figure 2a, the highlighted cell can be seen to transit from crawling to walking over 30 min. Analyzing the orientation manually for all tracks on glass indicates that out of 353 cells 166 exhibited walking at some point in the 60 min that they were observed, some transitioning to walking from crawling on multiple occasions, but a slight majority of cells were only observed to crawl (Figure S7). As a proxy readout for these modes, we used the degree of tilting that we measured automatically using an image analysis script for all of the samples, as previously described by Rodesney et al.,^36^ to assign the twitching mode based on the aspect ratio of the cells. A newly divided individual cell lying flat on a surface has an aspect ratio of 2, as seen in the leftmost frame of Figure 2a. When the aspect of cell is <2, the cell can be described as “tilting” (Figure 2a, right side). Aspect ratios >2 denote horizontal cells. This may also include cells undergoing division that are in contact with the surface at only one pole in a tilting position. Visual inspection of all frames showed that the fraction of cells undergoing division was <2% and the contribution of this error was evaluated as low. The fraction of cells that are tilting, the “tilting fractions”, for all polymers is shown in Figure 3, ranging from 0.17 for pPhEMA to 0.34 for pHPhOPA of cells in a tilted orientation at time points sampled. A linear correlation (*R*^2^ = 0.56) exists between twitching speed and tilting fraction on all materials excluding one outlier, poly(furfuryl methacrylate) pFuMA that showed an absolute residual greater than 2 × SD of the speed. This indicated that cells in a walking orientation moved faster. This observation is consistent with the higher number of surface interactions in the parallel crawling orientation reducing speed and more efficient surface–T4P traction in the walking orientation increasing speed. Rodesney et al. suggested in their work on glass surfaces that there was a friction-like force between the cells and the surface originating from the biomolecules coating the cell body that reduced speed in the parallel (crawling) orientation.^36^

### Surface Physical Properties and Bacterial Twitching Speed

Bacterial behavior such as bacterial attachment to surfaces has previously been shown to respond to both roughness and WCA on poly(dimethylsiloxane) (PDMS) surfaces and on two thermally switchable acrylate polymers.^39,40^ To assess the influence of polymer surface roughness on twitching speed, we used AFM to measure the RMS roughness for the polymers. This ranged from a low of 0.2 nm for poly(ethylhexyl methacrylate) pEHMA to a high of 1.4 nm for poly(ethylene glycol dimethacrylate) pEGDMA (Figure 1d). The twitching speed exhibited a negligible correlation with surface roughness (Figure S8). T4P have an outer diameter of approximately 5–8 nm and vary in length from 0.5 to 7 μm, much larger than the measured polymer RMS roughness.^41^ Thus, it appears unlikely that polymer topography affects bacterial motility. Physicochemical properties such as polymer wettability and stiffness were also considered. No significant correlations were observed between twitching speed and polymer WCA or Young’s modulus (Figure S8). This is likely due to the use of a broader range of chemistries adopted in this work compared to those in previous works.^27^ Estimates of *P. aeruginosa* cell wall stiffness are between 100 and 200 MPa, which are 3 orders of magnitude lower than Young’s moduli measured with our polymers.^42^ A positive correlation between material stiffness and *P. aeruginosa* attachment, and intracellular secondary messenger cyclic dimeric guanosine monophosphate (c-di-GMP) has been previously observed for silicone substrates measured to have bulk moduli measured in the range of 0.1–2.6 MPa.^43^ C-di-GMP is a key regulator of biofilm formation and is known to negatively regulate bacterial motility.^44^ Thus, our lack of a correlation between stiffness and bacterial twitching speed suggests that it is likely that the stiffness range explored in our study (1.8–4.3 GPa in wet conditions) is greater than that to which *P. aeruginosa* can respond.

### *P. aeruginosa* Biofilm Formation

**Figure 4:**
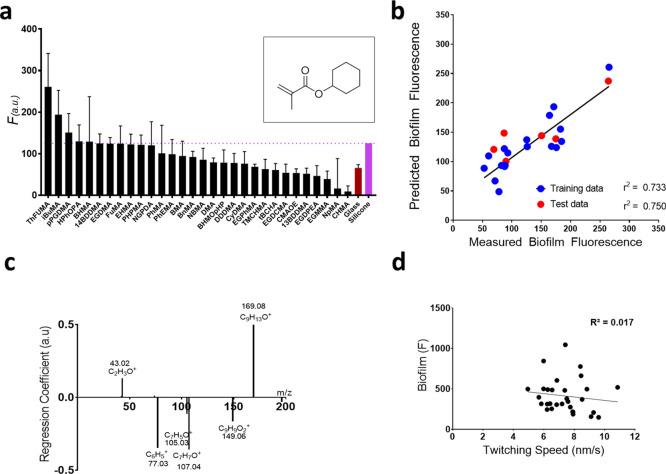
(a) *P. aeruginosa* biofilm formation measured as fluorescence (*F*) on each polymer after 24 h incubation. Error bars show ±1 SD (*N* = 3). Glass and silicone samples are identified as red and violet, respectively, for comparison. The inset shows pCHMA monomer structure; (b) a PLS regression model was used to predict the *F* value using the ToF-SIMS spectra for the polymers (*R*^2^ = 0.73 and 0.75, RMSE = 15.41 and 13.48 for the training and test data sets, respectively); (c) the molecular ion regression coefficients (RCs) relating to high and low biofilm formations, respectively; and (d) scatter plots showing the lack of correlation between biofilm formation (*F*) and twitching speed.

T4P and twitching motility make important contributions to the different stages of *P. aeruginosa* biofilm development (initial surface interactions, subsequent microcolony formation, structuring of biofilm architecture), albeit in a growth environment-dependent manner.^7^ Thus, we explored whether a relationship existed between twitching speed and biofilm formation for the library of polymers. To assess biofilm formation, *P. aeruginosa* was tagged with a fluorescent protein (mCherry) and inoculated into an RPMI-1640 medium containing the polymer microarray. Biofilm formation on each polymer spot was quantified *via* the fluorescence readout after 24 h incubation at 37 °C compared to the biofilm formed on the control materials, glass and silicone surfaces (Figures 4a and S9). The fluorescence intensity has been shown previously to correlate highly with the number of bacteria. Biofilm formation differed across the polymers and on silicone and glass. Under these conditions, poly(tetrahydrofurfuryl methacrylate) pThFUMA showed the highest fluorescence value, almost twice as high as silicone. The lowest fluorescence value, 93% smaller than silicone, was for pCHMA (Figure 4a).

To determine the relationship between polymer surface chemistry and *P. aeruginosa* biofilm formation, ToF-SIMS data and chemometrics analysis were employed. LASSO was again performed to create a sparse data set with uninformative features removed and 10 features considered (Table S2). A PLS model was constructed using the training set (75% of the polymers) and was validated using a test set (25% of the polymers). Biofilm formation was successfully predicted for the polymers in the training set (*R*^2^ = 0.75, RMSE = 15.4; Figure 4b) and test set (*R*^2^ = 0.73, RMSE = 13.5), demonstrating a good predictive performance for the model and lack of over-fitting. For the polymer library studied, this model shows that surface chemistry, as described by ToF-SIMS ion peaks, strongly influences biofilm formation, consistent with earlier studies.^45^ The biofilm formation model derived from the ToF-SIMS data showed a marginally stronger correlation than models reported in our previous work (*R*^2^ = 0.68).^22^ This is likely to be a consequence of the smaller number of materials used. The influence of each molecular ion on bacterial biofilm formation was described by size and sign of the PLS regression coefficients, where a positive coefficient indicates that the ion in question was associated with high biofilm formation and a negative indicates resistance to biofilm (Figure 4c). The phenyl secondary ion ([C_6_H_5_]^+^) and oxygen-conjugated O-benzyl (phenyl ether) groups ([C_7_H_5_O^+^] and [C_7_H_7_O^+^]) were associated with low *P. aeruginosa* biofilm formation, consistent with previous observations.^21,22,25^ Oxygen-containing ions from specific pendant groups such as ethylene glycol ([C_2_H_3_O]^+^) were associated with relatively high biofilm formation for this library.

Despite clear relationships between polymer surface chemistry and biofilm formation and twitching speed individually, no clear correlation was found between biofilm formation and twitching speed for the polymer library (Figure 4d). This is exemplified by pHPhOPA and pCHMA, both of which induced fast twitching speeds (Figure 2b). However, pCHMA exhibited very low biofilm formation, while pHPhOPA was associated with relatively high biofilm formation (Figure 4a). Interestingly, p*t*BCHA induced both relatively low biofilm formation and low twitching speed. This difference is likely to be due to the presence of a hydroxyl group in pHPhOPA, as we previously observed hydroxyl groups playing a key role in supporting bacterial biofilm formation.^21,45^ In addition, no correlations were found between biofilm formation and the fraction of nonmotile cells on each polymer or between biofilm formation and the accumulation rates of cells on the polymer surfaces during the first hour of exposure to surfaces (Figure S10a,b), obtained from the difference between on/off cell rates (Figure S6). This reinforces our findings that twitching speed and biofilm formation depend on different surface chemistries (Table S3) and leads us to hypothesize that changes in bacterial dispersal at later time points may explain diverse biofilm formation on polymers. The three diacrylates poly(1,3-butanediol dimethacrylate) p13BDDMA, pDDDMA, and p14BDDMA, all of which contain an aliphatic carbon linker group, exhibited disparate biological responses. For example, the second-fastest and lowest twitching speeds were observed on p13BDDMA and p14BDDMA, respectively, despite these polymers having a similar WCA and roughness. This suggests that bacteria are able to respond to subtle different surface chemistries alone. Previous assessment of bacterial attachment and biofilm formation to a polymer microarray also demonstrated that quite subtle changes in polymer chemistry such as regioisomers significantly altered the responses of bacteria.^21^ Thus, surface chemistry affects the initial bacteria–surface interactions associated with twitching and the later-stage biofilm formation in markedly different ways. Twitching motility has been shown to contribute to cell aggregation and microcolony formation during early-stage biofilm development (later than 1 h of incubation).^7^ Thus, we explored biofilm architecture on two polymers that were found to both promote biofilm and exhibit disparate twitching speeds (pHPhOPA high and pNGPDA moderate). As shown in Figure S11, slightly larger aggregates were observed on pHPhOPA compared to those on pNGPDA. Although no correlation has been found between twitching and biofilm, the different biofilm morphologies on different spots suggest that the surface chemistries affecting twitching may still play a role in influencing the biofilm formation at a later time point. Surface interactions associated with biofilm formation are complex, involving multiple sensing mechanisms.^17,46^ The genome of *P. aeruginosa* contains ∼6000 genes, around 10% of which are devoted to environmental sensing and adaptation, and includes over 60 sensor regulator pairs, riboregulators, and second messengers.^47^ Multiple external signals are thus sensed, received, and integrated through sophisticated gene regulatory networks to instruct cell behavior.^46^ Surface sensing involved with twitching is likely to involve not just pili, although speed may also be determined by physicochemical interactions between the membrane and the surface. For this reason, it is not surprising that the surface chemistries involved with the twitching and biofilm formation responses are different.

Bacterial surface interactions generally are complex, and our results suggest that *P. aeruginosa* twitching and biofilm formation cannot be explained by material hydrophobicity and surface compliance alone, an observation consistent with the literature.^27^ However, the biological responses of twitching speed and biofilm formation individually can be correlated with surface chemistry. We observed materials with similar side-chain chemistry but different stiffness (*e.g*., pCHMA and p*t*BCHA), exhibiting similar biofilm formation but different twitching speeds, while materials with similar stiffness (*e.g*., pHPhOPA and poly(ethylene glycol phenyl) methacrylate pEGPhMA) but only subtly different surface chemistries giving different biological responses (Figure S12). Thus, although stiffness, porosity, and physical structure likely do alter biological response,^43,48^ for the polymers studied here, surface chemistry appears to be the key influence on biological response, possibly due to the fact that the stiffness range explored was outside that which *P. aeruginosa* responds to.

## Conclusions

A microarray of 30 methacrylate/acrylate polymers was generated by photoinduced free-radical polymerization on a glass support optimized for *in situ* optical microscopy. The microarray was studied by high-resolution time-lapse microscopy to assess bacterial twitching motility on each polymer surface to elucidate the initial bacterial surface responses at the single-cell level. Surface characterization data was used to train chemometrics models that generated predictive, linear relationships between surface chemistry and both average twitching speed and biofilm formation. Twitching speed and biofilm formation were not significantly correlated across the library, suggesting that the two biological processes are determined by different surface chemistry–bacterial interactions. In terms of biofilm, this suggests that the chemical moieties that determine bacterial behavior after initial reversible attachment differ from those associated with the transition to irreversible attachment. This is likely because of the differing roles of the bacterial sensing mechanisms (flagella, T4P, and the cell membrane) at these different stages. This insight elucidates how material surfaces influence bacterial behavior and will aid in the development of novel polymers that influence bacteria–surface interactions at both early and late stages of biofilm formation.

## Supporting Information Available

Names, structures, and acronyms of the monomers; list of ToF-SIMS ions associated with biofilm data and twitching speed obtained from LASSO test; list of the dominant descriptors for each predictive models; schematic of polymer microarray production using contact printing; schematic of bespoke inverted microscope holder for bacterial culture polymer microarray; scatter plot comparing the difference of Young’s moduli under dry and liquid conditions and clogP for each polymer; representative colormap of *P. aeruginosa* wild-type and *pilA* mutant twitching motility; Gaussian distribution of twitching speed on polymers; twitching motility speed does not depend on the number of surface-associated cells; characterization of different orientations that *P. aeruginosa* motile cells adopted relative to glass surface; scatter plots comparing bacterial speed with WCA, Young’s modulus, and RMS deviation; the fluorescence *F* value as a measurement of *P. aeruginosa* biofilm formation on silicone; scatter plots showing the lack of correlation between biofilm formation and both fraction of nonmotile cells and on–off cell rate; confocal images of *P. aeruginosa* biofilm on two exemplar polymeric surfaces; and heatmap of polymer stiffness, biofilm formation, and twitching speed (PDF)

## Notes

All relevant data are available from the University of Nottingham’s Research Data Management Repository.
