# Supplementary material for: Single-Cell Tracking on Polymer Microarrays Reveals the Impact of Surface Chemistry on Pseudomonas aeruginosa Twitching Speed and Biofilm Development
Source: ACS Appl Bio Mater. 2020 Nov 6;3(12):8471–80. doi: 10.1021/acsabm.0c00849 (PMC8291582; doi:10.1021/acsabm.0c00849)
Supplement: Supplementary file 1 — mt0c00849_si_001.pdf [file mt0c00849_si_001.pdf]

SUPPORTING INFORMATION

Single cell tracking on polymer microarrays reveals the impact of surface chemistry on *Pseudomonas aeruginosa* twitching speed and biofilm development

Alessandro M. Carabelli[1], Marco Isgró[1], Olutoba Sanni[1], Grazziela P. Figueredo[2], David A. Winkler[1,5,6,7], Laurence Burroughs[1], Andrew J. Blok[3], Jean-Frédéric Dubern[4], Francesco Pappalardo[1], Andrew L. Hook[1], Paul Williams[4] and Morgan R. Alexander[1]**

[1]Advanced Materials and Healthcare Technologies, School of Pharmacy, University of Nottingham, Nottingham NG7 2RD, UK; [3]School of Computer Science, University of Nottingham, Nottingham NG8 1BB, UK; [3]Division of Molecular Therapeutics and Formulation, School of Pharmacy, University of Nottingham, Nottingham, NG7 2RD, UK; [4]Biodiscovery Institute and School of Life Sciences, University of Nottingham, Nottingham NG7 2RD, UK; [5]Monash Institute of Pharmaceutical Sciences, Monash University, Parkville, Australia; [6]La Trobe Institute for Molecular Science, la Trobe University, Bundoora, Australia; [7]CSIRO Data61, Pullenvale 4069, Australia.

*Joint corresponding authors

Contact e-mail: morgan.alexander@nottingham.ac.uk and paul.williams@nottingham.ac.uk

Table S1. Names, acronyms and structures of the monomers.

#	Name	Structure	Acronym	Source or reference
1	1,10-Decanediol dimethacrylate	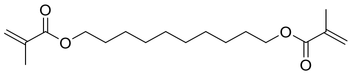	DDDMA	This study
2	Decyl methacrylate	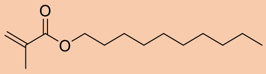	DMA	[1]
3	2,2-Bis[4-(2-hydroxy-3-methacryloxypropoxy) phenyl]propane	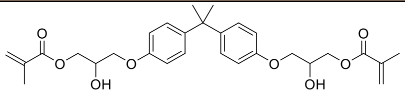	BHMOPhP	This study
4	Isobutyl methacrylate	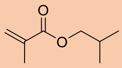	iBuMA	[1]
5	Hydroxy-3-phenoxypropyl acrylate	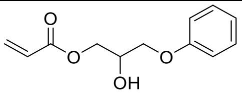	HPhOPA	This study
6	Caprolactone 2-(methacryloyloxy)ethyl ester	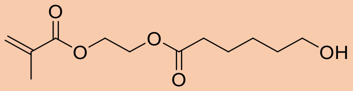	CMAOE	[1]
7	Hydroxypropyl methacrylate	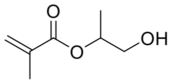	HPMA	[1]
8	Ethylene glycol dimethacrylate	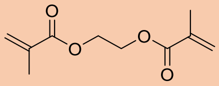	EGDMA	This study
9	Cyclododecyl methacrylate	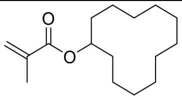	CyDMA	[2]
10	Cyclohexyl methacrylate	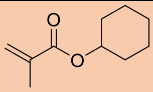	CHMA	[1-2]
11	Ethylene glycol phenyl methacrylate	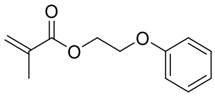	EGPhMA	This study
12	1,3-Butanediol dimethacrylate	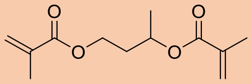	13BDDMA	This study
13	Tetrahydrofurfuryl methacrylate	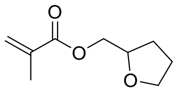	THFuMA	[1]
14	Ethylene glycol dicyclopentenyl ether methacrylate	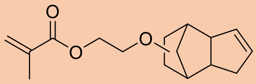	EGDCMA	[1]
15	Benzyl methacrylate	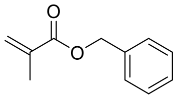	BnMA	[1, 3]
16	1,4-Butanediol dimethacrylate	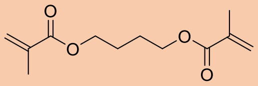	14BDDMA	This study

17	Tert-butylcyclohexylacrylate	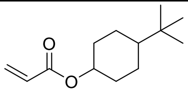	tBCHA	[4]
18	2-Phenylethyl methacrylate	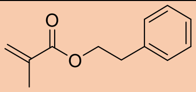	PhEMA	This study
19	Butyl methacrylate	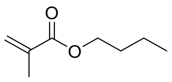	BMA	[1]
20	Phenyl methacrylate	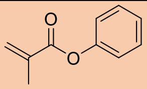	PhMA	[1]
21	Norbornyl methacrylate	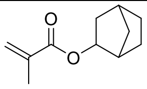	NBMA	[1]
22	Furfuryl methacrylate	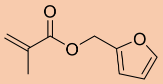	FuMA	[1]
23	Poly(propylene glycol) (400) dimethacrylate	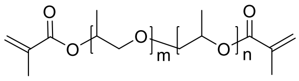	pPGDMA	This study
24	3-Phenoxy 2 hydroxy propyl methacrylate	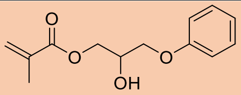	PHPMA	This study
25	Ethylene glycol methyl ether methacrylate	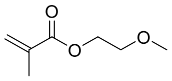	EGMMA	[1]
26	Trimethylcyclohexyl methacrylate	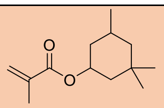	TMCHMA	[1]
27	Ethylhexyl methacrylate	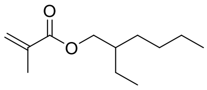	EHMA	[1]
28	Benzhydryl methacrylate	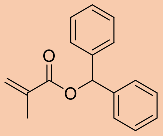	BHMA	This study
29	Neopentyl glycol propoxylate diacrylate	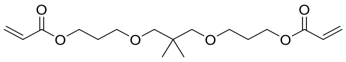	NGPDA	[1]
30	Ethylene glycol dicyclopentenyl ether acrylate	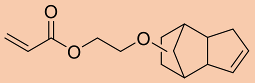	EGDPEA	[4]

Table S2. List of ToFSIMS ions associated with biofilm data **(a)** and twitching speed **(b)** obtained from LASSO test.

a

m/z	assignment
42.05	$[C_3H_6]^-$
43.02	$[C_2H_3O]^+$
74.00	$[C_2H_2O_3]^+$
77.03	$[C_6H_5]^+$
105.03	$[C_7H_5O]^+$
107.04	$[C_7H_7O]^+$
149.04	$[C_{12}H_5]^-$
149.06	$[C_9H_9O_2]^+$
169.08	$[C_9H_{13}O_3]^+$
196.07	$[C_{10}H_{12}O_4]^+$

b

m/z	assignment
25.01	$[C_2H]^-$
29.01	$[CHO]^-$
62.02	$[C_5H_2]^-$
83.08	$[C_6H_{11}]^+$
88.03	$[C_7H_4]^-$
133.07	$[C_9H_9O]^-$
155.14	$[C_{10}H_{19}O]^-$
175.06	$[C_{11}H_{11}O_2]^-$
178.06	$[C_{10}H_{10}O_3]^-$

Table S3. List of the dominant descriptors by regression coefficients (RC) for predictive models associated to twitching motility **(a)** and biofilm formation **(b)** obtained using PLS regression.

a

Pro- twitching motility

m/z	assignment	RC
155.14	$[C_{10}H_{19}O]^-$	0.77
133.07	$[C_9H_9O]^-$	0.11
175.06	$[C_{11}H_{11}O_2]^-$	0.08
88.03	$[C_7H_4]^-$	0.04

Anti- twitching motility

m/z	assignment	RC
62.02	$[C_5H_2]^-$	-0.09
83.08	$[C_6H_{11}]^+$	-0.07

b

Pro- biofilm

m/z	assignment	RC
169.08	$[C_9H_{13}O_3]^+$	0.83
43.02	$[C_2H_3O]^+$	0.13

Anti- biofilm

m/z	assignment	RC
107.04	$[C_7H_7O]^+$	-0.36
77.03	$[C_6H_5]^+$	-0.35
149.06	$[C_9H_9O_2]^+$	-0.16
105.03	$[C_7H_5O]^+$	-0.11

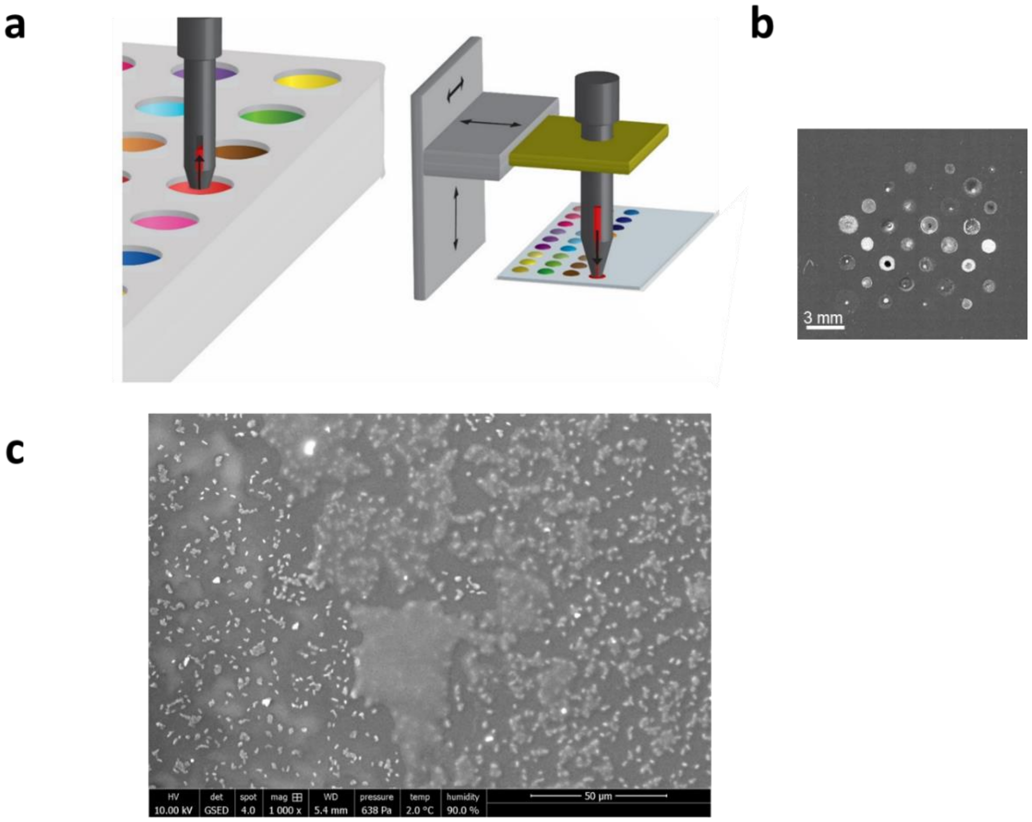

Figure S1. (a) Schematic of polymer microarray production using contact printing. Spotted monomer solutions, shown in different colors are transferred from a multi-well source plate (left) to the slide surface (right). A quilled metal pin was used in this study; **(b)** Phase Contrast microscope image of the microarray. Scale bar, 3 mm**. (c)** ESEM representative image of PAO1 grown for 24 h in RPMI-1640 on a pro-biofilm polymer. Image captured at 1,000× magnification.[i]

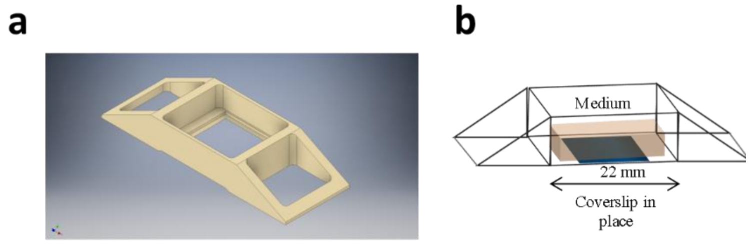

Figure S2. (a) Schematic of bespoke inverted microscope holder for bacterial culture polymer microarrays; (**b)** The 22 × 22 mm coverslip was sealed at the bottom using a silicone- and halogen-free high-vacuum grease (Apiezon M).

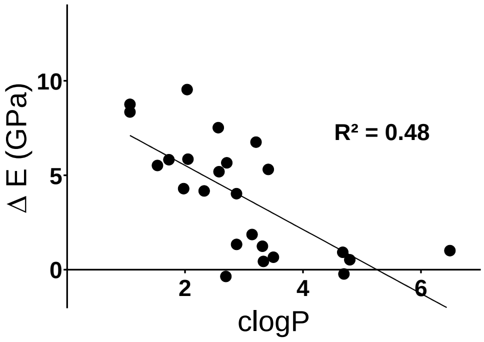

Figure S3. Scatter plot comparing the difference of Young's moduli under dry and liquid conditions for each polymer (ΔE) and hydrophilicity of the polymer shown as clogP (R^2 = 0.48).

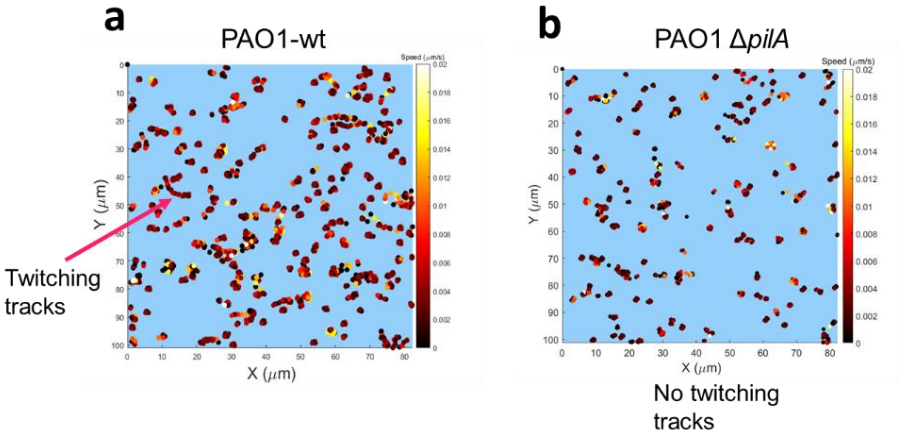

Figure S4. Representative colormap of *P. aeruginosa* wild type **(a)**, and *pilA* mutant **(b)** twitching on a glass surface obtained from images acquired every 2 minutes for 1 hour. The absence of tracks in (**b**) are consistent with the requirement of T4P pili for twitching motility. Brighter coloured (yellow) dots are Δ*pilA* cells spinning on the surface attached via their flagella.

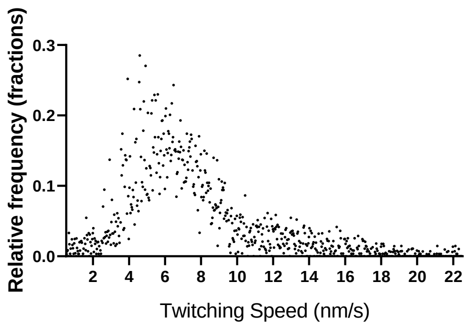

Figure S5. Gaussian distribution of twitching speed on all polymers. Scatter plot shows the frequencies of instantaneous *P. aeruginosa* twitching cell speeds (bin=1) on each polymer.

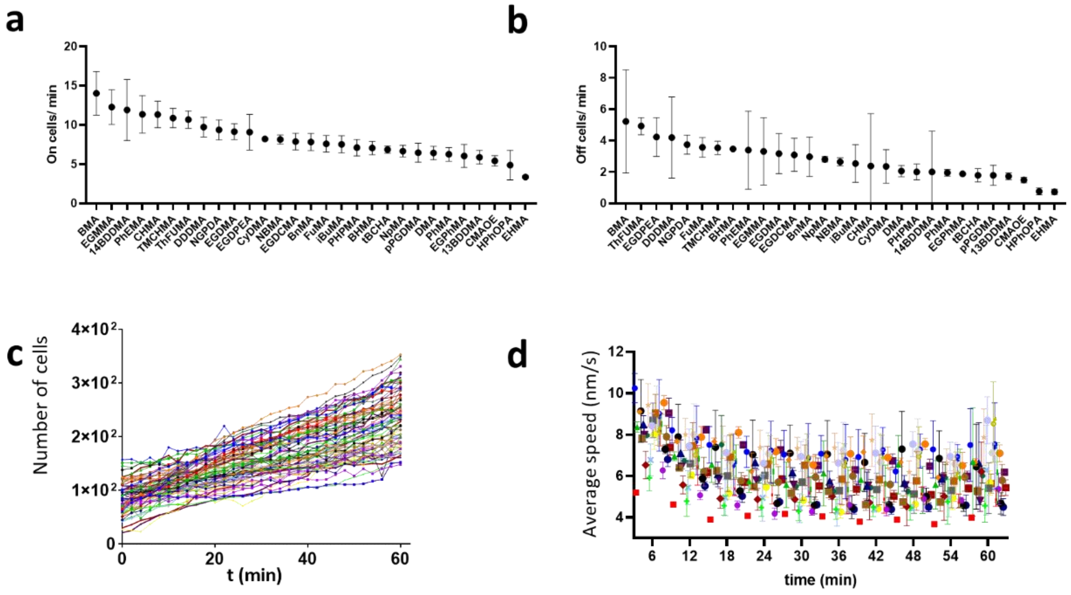

Figure S6. Twitching motility speed does not depend on the number of surface-associated cells. **(a)** Average on and **(b)** off rates (number of cells/ min) of bacteria on each polymer over the 1 hour of incubation. (N=3) On rates consider both cells arrivals and doubling; **(c)** Number of cells per frame on each polymer. Colors show different polymers; **(d)** average twitching speed of *P. aeruginosa* cells on each polymer at different intervals (every 6 minutes) (N=3).[ii]

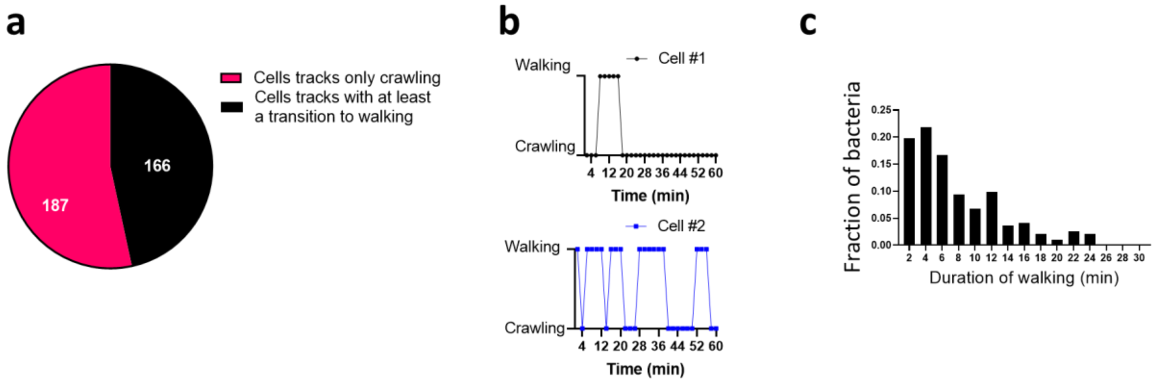

Figure S7. Characterization of different orientations that *P. aeruginosa* motile cells adopted relative to glass surface over 1 hour from the inoculation. **(a)** Pie chart shows the number of cells tracks crawling only (cells moving with the cell body parallel to the surface) (in pink) and cells with transition to walking (tilted and attached to surface on one pole) (in black). n = 353; **(b)** Representative transitions from crawling to walking of 2 cells over time; **(c)** Histogram shows the fraction of bacteria that exhibited at least one walking event *vs* duration of walking (min) after recording substrate inoculum (n = 166).

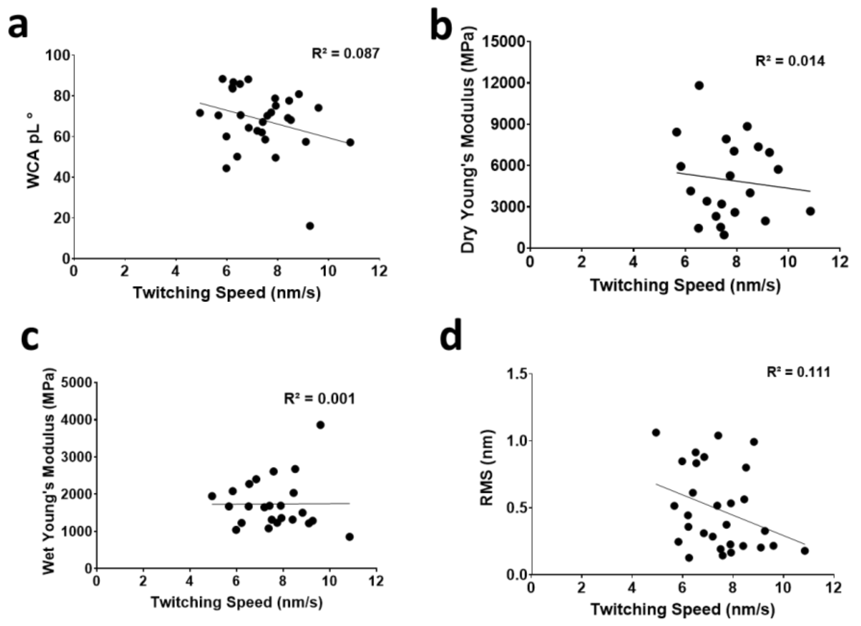

Figure S8. Scatter plots comparing bacterial speed with WCA **(a)**, Young's modulus in dry **(b)** and wet conditions **(c)** and RMS deviation **(d)**. No correlation was found using the entire library of materials.

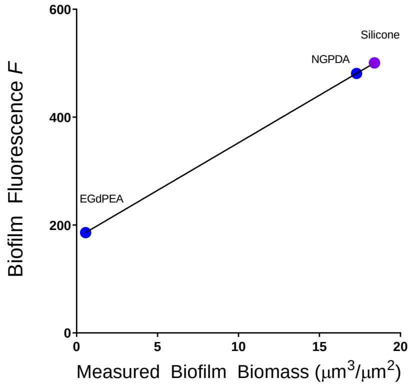

Figure S9. The *F* value as a measurement of *P. aeruginosa* biofilm formation on silicone (violet dot) was inferred from *F* values for pNGPDA and pEGdPEA, reference polymers with respectively biofilm promoting and biofilm inhibitory properties. The line was fitted from the biofilm biomass data obtained from Dundas *et al.*[2]

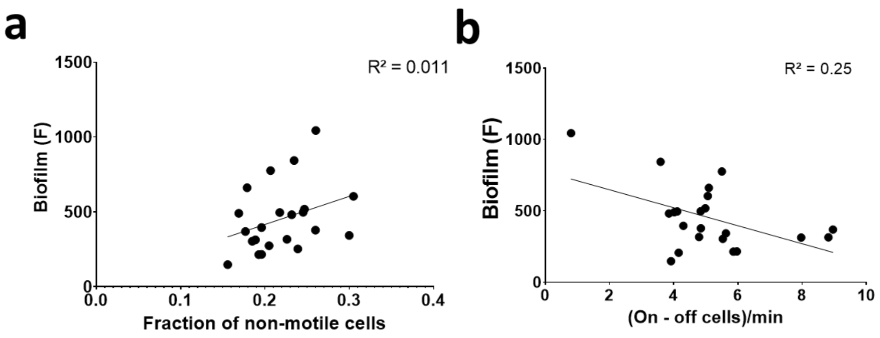

Figure S10. Scatter plots show the lack of correlation between biofilm formation (*F*) and **(a)** fraction of non-motile cells and **(b)** the average cell accumulation rate as difference between on and off cells/ min (N=3).

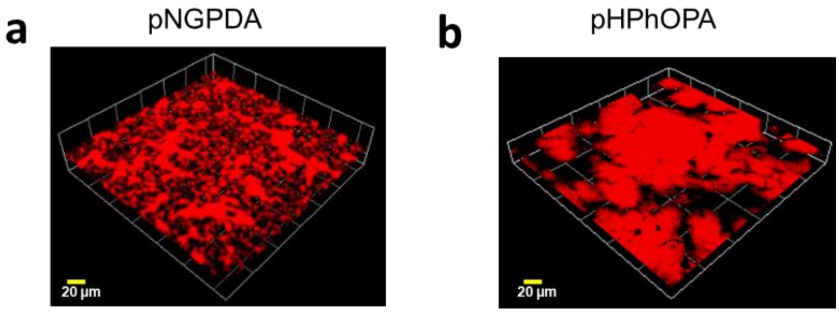

Figure S11. Confocal images (10×, 0.3) of *P. aeruginosa m-cherry* tagged PAO1-W after 24h on two exemplar polymeric surfaces pNGPDA **(a)** and pHPhOPA **(b)**. Samples were washed 2× in PBS and 1× in H_2O. Scale bar is 20 µm.[iii]

	Young's modulus (wet conditions) (GPa)	Biofilm (F) (a.u)	Twitching speed (nm/min)
CHMA	3.9 ± 1.1	9.0 ± 23.6	11.0 ± 2.4
BnMA	2.7 ± 1.1	92.3 ± 23.6	10.9 ± 2.5
NBMA	2.6 ± 1.0	85.5 ± 48.0	10.8 ± 2.4
CyDMA	2.4 ± 0.9	76.0 ± 50.4	7.9 ± 1.1
PhPMA	2.3 ± 0.6	121.5 ± 40.5	8.4 ± 1.5
PhMA	2.0 ± 0.7	101.0 ± 89.0	9.7 ± 1.3
EGDMA	1.9 ± 0.3	124.3 ± 25.4	7.4 ± 1.6
EGDCMA	1.7 ± 0.4	54.0 ± 36.2	9.7 ± 1.5
THFUMA	1.7 ± 0.4	261.0 ± 138.9	9.2 ± 1.3
TMCHMA	1.7 ± 1.4	63.0 ± 40.5	7.8 ± 0.8
PhEMA	1.7 ± 0.5	98.8 ± 61.3	7.1 ± 0.6
NpMA	1.7 ± 1.0	16.5 ± 124.3	8.9 ± 1.2
FUMA	1.5 ± 0.4	124.0 ± 74.0	10.3 ± 2.2
EGDPEA	1.4 ± 0.3	46.5 ± 42.8	8.8 ± 1.4
iBuMA	1.3 ± 0.6	194.0 ± 101.2	9.6 ± 1.6
BMA	1.3 ± 0.3	94.5 ± 61.7	10.2 ± 1.9
13BDDMA	1.3 ± 0.4	51.8 ± 23.3	11.2 ± 2.9
EGPhMA	1.2 ± 0.3	68.5 ± 10.7	8.7 ± 1.4
tBCHA	1.2 ± 0.6	60.8 ± 27.5	7.0 ± 0.9
EGMMA	1.2 ± 0.4	39.0 ± 33.3	10.2 ± 1.7
NGPDA	1.1 ± 0.1	120.3 ± 98.0	9.9 ± 2.1
14BDDMA	1.0 ± 0.1	124.8 ± 39.3	6.1 ± 0.5
HPhOPA	0.9 ± 0.2	129.5 ± 69.0	12.9 ± 1.5

Figure S12. Heatmap of polymers Young's modulus (in wet conditions), *P. aeruginosa* biofilm formation (*F*) and average twitching speed associated to each polymer (red indicating high values and white low values). Values are ranked in descending order for stiffness. The center square is the average value, whilst the narrow columns to the right indicate ± 1 sd unit respectively (N=3).

REFERENCES

(1) Hook, A. L.; Chang, C.-Y.; Yang, J.; Atkinson, S.; Langer, R.; Anderson, D. G.; Davies, M. C.; Williams, P.; Alexander, M. R. Discovery of novel materials with broad resistance to bacterial attachment using combinatorial polymer microarrays. *Advanced Materials* **2013**, *25* (18), 2542-2547.
(2) Dundas, A. A.; Sanni, O.; Dubern, J.-F.; Dimitrakis, G.; Hook, A. L.; Irvine, D. J.; Williams, P.; Alexander, M. R. Validating a predictive structure–property relationship by discovery of novel polymers which reduce bacterial biofilm formation. *Advanced Materials* **2019**, *31* (49), 1903513.
(3) Sanni, O.; Chang, C.-Y.; Anderson, D. G.; Langer, R.; Davies, M. C.; Williams, P. M.; Williams, P.; Alexander, M. R.; Hook, A. L. Bacterial attachment to polymeric materials correlates with molecular flexibility and hydrophilicity. *Advanced Healthcare Materials* **2015**, *4* (5), 695-701.
(4) Hook, A. L.; Chang, C.-Y.; Yang, J.; Luckett, J.; Cockayne, A.; Atkinson, S.; Mei, Y.; Bayston, R.; Irvine, D. J.; Langer, R.; Anderson, D. G.; Williams, P.; Davies, M. C.; Alexander, M. R. Combinatorial discovery of polymers resistant to bacterial attachment. *Nature Biotechnology* **2012**, *30* (9), 868-875.
(5) Williams, P. Quorum sensing, communication and cross-kingdom signalling in the bacterial world. *Microbiology (Reading, England)* **2007**, *153* (Pt 12), 3923-3938.

---

[i] Environmental scanning electron micrographs were obtained using a Quanta 200FEG ESEM (FEI Company, Eindhoven, the Netherlands). Samples of polymers coupons incubated with *P. aeruginosa* were glued to the holders using die-cut carbon conductive adhesive discs (SPI Supplies/Structure Probe, Inc., West Chester, PA, USA). Image is obtained using Peltier Stage and gaseous secondary electron detector and captured at 1,000× magnification. The chamber parameters were settled to 2°C temperature and 600 Pa pressure, at 10-15 kV. With these parameters the samples retain 85% of relative humidity. Then the chamber parameters were settled to <-10 °C temperature and 238 Pa pressure, at 5-15 kV in order to progressively sublimate the water content of the sample.

[ii] Bacterial populations alter their behaviour in response to population density.[5] Bacterial density is the result of doubling, arrivals and dispersions events. Different cell on and /off rates on each polymers were observed over the first hour of incubation with surfaces (**Figure S6a-b**). Thus, we investigated the growth rates on different polymers to assess whether the variable twitching speeds were caused by changes to population density. Cell population density increased over time on all polymers but no statistical differences were observed between samples (**Figure S6a**). To assess whether twitching speed was dependent on incubation time, we assessed bacterial speeds for each polymer at 6 minutes intervals over the first hour of exposure to polymers. As shown in **Figure S6b**, average twitching speeds were constant over this time period for all polymers. This suggests that neither surface exposure time nor changes in surface bacterial population cell-density influenced the speed of twitching.

[iii] The polymers coated slides were examined directly under a Laser Scanning fluorescent Microscope (LSM2, Zeiss) using mCherry mode at an excitation wavelength of 587nm. Imaging was carried out using Zen 2011 imaging software (Zeiss). A total of 10 Z-stacked images were collected per polymer spot.
